# PEDV ORF3 Independently Regulates IκB Kinase β-Mediated NF-κB and IFN-β Promoter Activities

**DOI:** 10.3390/pathogens9050376

**Published:** 2020-05-14

**Authors:** Challika Kaewborisuth, Surapong Koonpaew, Kanjana Srisutthisamphan, Ratchanont Viriyakitkosol, Peera Jaru-ampornpan, Anan Jongkaewwattana

**Affiliations:** 1 Virology and Cell Technology Laboratory, National Center for Genetic Engineering and Biotechnology (BIOTEC), National Science and Technology Development Agency (NSTDA), Pathumthani 12120, Thailand; challika.kae@biotec.or.th (C.K.); surapong.koo@biotec.or.th (S.K.); kanjana.sri@biotec.or.th (K.S.); peera.jar@biotec.or.th (P.J.-a.); 2 Faculty of Veterinary Science, Chulalongkorn University, Bangkok 10330, Thailand; ratchanont.v@student.chula.ac.th

**Keywords:** ORF3, Porcine epidemic diarrhea virus, IKBKB, NF-κB, IFN-β, Type I IFN induction

## Abstract

The Open Reading Frame 3 (ORF3), an accessory protein of porcine epidemic diarrhea virus (PEDV), has been shown to interact with a myriad of cellular proteins, among which include the IκB kinase β (IKBKB). Here, specific IKBKB domains responsible for ORF3–IKBKB interaction were identified. Dysregulation of NF-κB and Type I interferon (IFN) in the presence of ORF3 was also demonstrated. We showed that while ORF3 was capable of up-regulating IKBKB-meditated NF-κB promoter activity, it surprisingly down-regulated the activation of IKBKB-meditated IFN-β promoter and expression of IFN-β mRNA. When overexpressed, ORF3 could suppress Poly I:C mediated type I IFN production and induction. Finally, we demonstrated that IKBKB- and RIG-I-mediated type I IFN induction by ORF3 resulted in different outcomes. Our study is the first to demonstrate the potential and complex roles of ORF3 in the involvement of aberrant immune signaling as well as in the virus–host interaction.

## 1. Introduction

Upon sensing virus entry through families of pathogen recognition receptors (PRRs), host cells unleash a wide range of signals, which culminate in the initiation of anti-viral machinery. The induction of type I interferons (IFNs), including IFN-αs and IFN-β, type III IFNs (IFN-λs), and prototypical proinflammatory cytokines, including interleukin-1 (IL-1) and tumor necrosis factor-α (TNF-α) are among the hallmarks of early host responses against invading viral pathogens. The nuclear factor kappa B (NF-κB) complex plays a key role in linking the presence of danger signals to the initiation of both inflammatory response and cellular anti-viral status [1,2]. Cellular localization of NF-κB depends on the activity of the inhibitor of kappa B kinase (IKK) complex. The IKK complex consists of two kinase subunits, IKKα (IKBKA) and IKKβ (IKBKB), and a regulatory subunit IKKγ/NF-κB essential modifier (NEMO). The IKBKB and NEMO are important for canonical NF-κB pathway regulation [3]. Phosphorylation of the inhibitor of NF-κB (IκB) by the IKK complex is required to allow NF-κB to be released from IκB and translocated into the nucleus to regulate the transcription of genes participating in host immune and inflammatory responses [4,5]. 

Porcine epidemic diarrhea virus (PEDV) is the major causative agent of severe watery diarrhea with very high mortality in neonatal piglets [6]. PEDV belongs to the *Alphacoronavirus* genus in the *Coronaviridae* family. Its positive-sense single-stranded RNA of approximately 28 kb in length encodes viral replicase polyprotein (pp) 1a and pp1ab; structural proteins: spike(S), envelope (E), membrane (M), and the nucleocapsid (N) proteins; and an accessory protein ORF3. We previously employed a proteomic-based approach to identify cellular proteins that potentially interact with the PEDV ORF3 protein [7]. Among many candidates, IKKβ, or IKBKB (IKK2), was identified as one of the ORF3 interacting partners [7]. Providing that other accessory proteins of other coronaviruses [8,9] have been shown to interfere with type I IFN and proinflammatory cytokine productions, it is of interest to examine whether PEDV ORF3 could likewise modulate immune signaling pathways especially through the IKBKB interaction. 

In this study, we investigated the potential roles of PEDV ORF3 in the host immune modulation. In particular, we hypothesized that ORF3 directly interacts with IKBKB, leading to the dysregulation of the NF-κB-mediated activity such as type I interferon (IFN) and proinflammatory cytokine productions. We demonstrated here that IKBKB-mediated NF-κB and IFN-β promoter activities were differently affected by the presence of ORF3. Our data not only underscore the role of ORF3 in manipulating the host’s immune response via IKBKB, but also provide further insights into our understanding of PEDV and host interaction.

## 2. Results

### 2.1. IKBKB Interacts with PEDV ORF3 and Inhibits PEDV Replication in VeroE6 Cells

**Figure 1:**
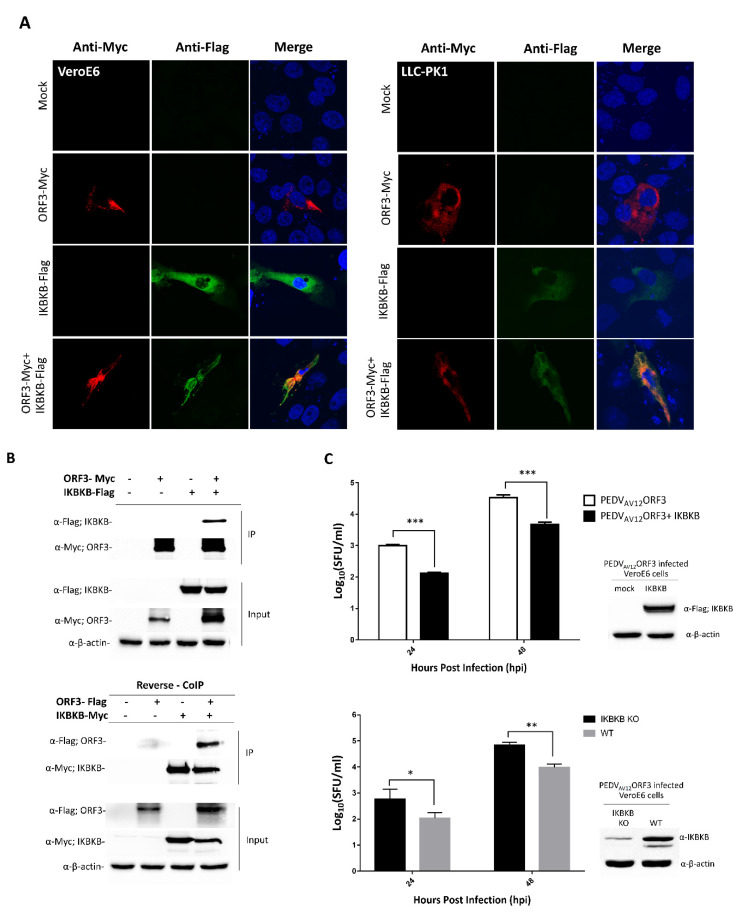
ORF3 and IκB kinase β (IKBKB) interaction and IKBKB’s effect on porcine epidemic diarrhea virus (PEDV) virus replication. (**A**) Indirect immunofluorescence confocal microscopy showing co-localization of ORF3 and IKBKB proteins in VeroE6 (left panel) and LLC-PK1 cells (right panel)**.** The plasmid expressing IKBKB with Flag-tag at the C-terminus was co-transfected with pCAGGS_ORF3-Myc in VeroE6 and LLC-PK1 cells. Cells transfected with empty plasmid was used as mock transfection. At 24 hpt, cells were incubated with mouse anti-Myc and rabbit anti-Flag antibodies. Goat anti-rabbit IgG Alexa flour 488 and goat anti-mouse IgG Alexa flour 647 were used as secondary antibodies. Protein localization was analyzed by confocal microscopy. (**B**) The plasmids expressing ORF3-Myc/ORF3-Flag and IKBKB-Flag/IKBKB-Myc proteins were co-transfected into HEK293T cells. The protein complexes were immunoprecipitated using anti-Myc bead. The immunoprecipitated proteins were probed with rabbit anti-Flag and anti-Myc antibodies. (**C**) Growth kinetics of the PEDV_AV12__ORF3 in overexpressed IKBKB- and IKBKB-knockout VeroE6 cells. VeroE6 (WT) cells, in triplicate, were transfected with a plasmid expressing IKBKB (upper panel). Cells transfected with empty vector were used as mock transfection control. At 8 hpt, cells were infected with PEDV_AV12__ORF3 at MOI of 0.1. Wild type (WT) and IKBKB knock-out (IKBKB KO) VeroE6 cells (lower panel), in triplicate, were infected with PEDV_AV12__ORF3 at MOI of 0.1. The virus (*n* = 3) from each group was harvested at 24 and 48 hpi and subjected to SFU for virus titration. Statistical analysis was performed by using the student t-test method. Error bars represent the means ± standard error of means of virus titers. *, *p* < 0.05; **, *p* < 0.01 and ***, *p* < 0.001.

Through a proteomic-based approach, we identified IKBKB as one of the ORF3 interacting partners in HEK293T cells [7]. Here, we showed that these two proteins indeed colocalized in both type I IFN incompetent VeroE6 and type I IFN competent LLC-PK1 cells (Figure 1A). Co-immunoprecipitation and reverse co-immunoprecipitation also confirmed that PEDV ORF3 protein strongly interacted with IKBKB (Figure 1B). As ORF3 has been proposed to play a role in regulating PEDV virus replication [10,11], we speculated that the overexpression of IKBKB might also affect PEDV replication. PEDV_AV12__ORF3 [12] replication was, therefore, determined in VeroE6 cells overexpressing IKBKB. As expected, we observed that the expression of IKBKB significantly suppressed PEDV replication in VeroE6 cells (Figure 1C). To assess whether the presence of endogenous IKBKB affects PEDV_AV12__ORF3 replication, IKBKB-knockout VeroE6 cells were generated and infected with PEDV_AV12__ORF3. In agreement with the results found in transfected cells, PEDV_AV12__ORF3 replication in IKBKB-knockout cells was more efficient than that in wild-type counterparts (Figure 1C).

### 2.2. Characterization of IKBKB and ORF3 Regions Responsible for IKBKB-ORF3 Binding

**Figure 2:**
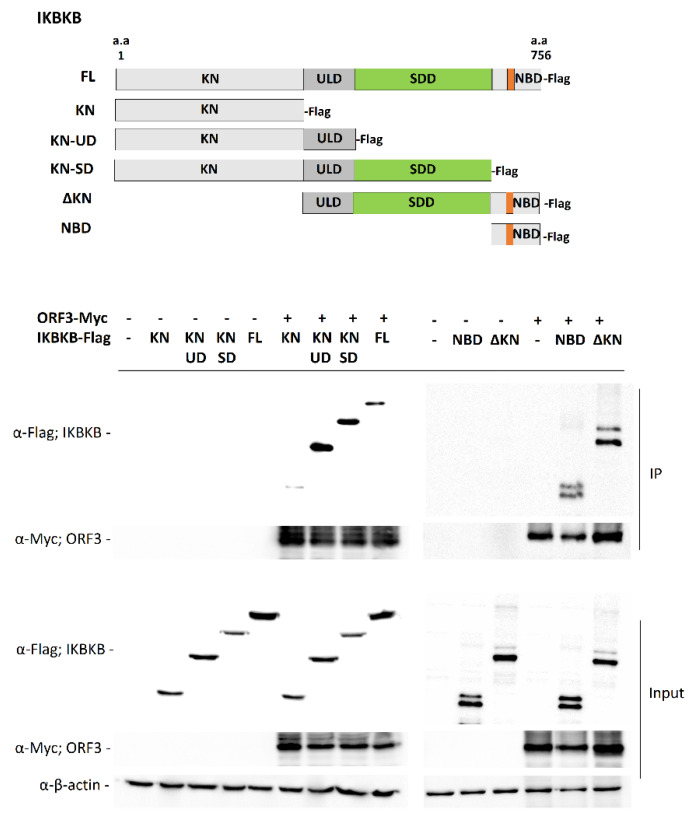
Detailed analysis of ORF3–IKBKB region interaction. Schematic representation showing the full-length IKBKB and those with truncated constructs (upper panel). The plasmid expressing ORF3-Myc was co-transfected with plasmids expressing each of truncated IKBKB with Flag-tag at C-terminus in HEK293T cells. At 24 hpt, cleared lysates were subjected to immunoprecipitation using anti-Myc beads. The immunoprecipitated proteins were probed with rabbit anti-Flag and anti-Myc antibodies. KN, Kinase domain; ULD, ubiquitin-like domain; SDD, scaffold/dimerization domain; NBD, NF-κB essential modifier (NEMO) binding domain; ΔKN, deleted kinase domain.

**Figure 3:**
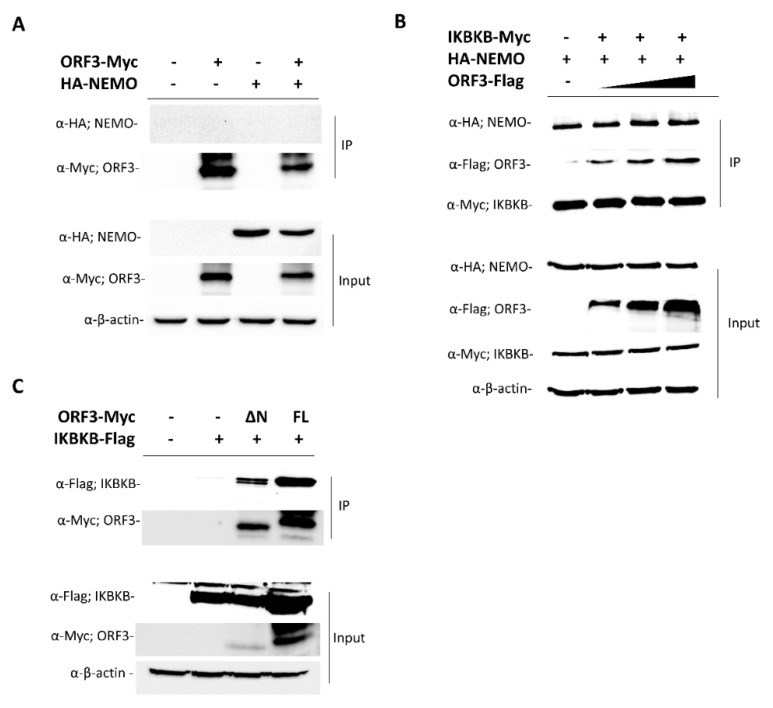
ORF3 interaction with NEMO and ORF3 regions–IKBKB interaction. (**A**) The plasmid expressing ORF3-Myc was co-transfected with plasmids expressing hemagglutinin tagged (HA)-NEMO in HEK293T cells. At 24 hpt, cleared lysates were subjected to immunoprecipitation using anti-Myc beads. The immunoprecipitated proteins were probed with rabbit anti-HA and anti-Myc antibodies. (**B**) Each of plasmids expressing IKBKB-Myc or HA-NEMO or ORF3-Flag was transfected in HEK293T cells. At 24 hpt, cleared lysates were collected. Equal amounts (0.4 mg/mL total protein) of lysates containing IKBKB-Myc and HA-NEMO and varied amounts (0.2, 0.4 and 0.8 mg/mL total proteins) of lysate with ORF3-Flag were combined and subjected to immunoprecipitation using anti-Myc beads. The immunoprecipitated proteins were probed with rabbit anti-Flag, anti-HA, and anti-Myc antibodies. (**C**) The plasmids expressing ΔN-ORF3-Myc (ΔN; lacking amino acids 1-73) and full length (FL; amino acids 1-224) ORF3-Myc were co-transfected with the plasmid expressing IKBKB-Flag in HEK293T cells. At 24 hpt, cleared lysates were subjected to immunoprecipitation using anti-Myc beads. The immunoprecipitated proteins were probed with rabbit anti-Flag and anti-Myc antibodies.

Structurally, IKBKB contains an N-terminal kinase domain (KN), a ubiquitin-like domain (ULD), an elongated α-helical scaffold/dimerization domain (SDD), and NEMO binding domain (NBD) [13]. To further investigate the ORF3-IKBKB interaction, a series of IKBKB truncation mutants were constructed to identify the IKBKB domain(s) responsible for IKBKB and ORF3 interaction (Figure 2). Each of these Flag-tagged truncated IKBKB domains was then co-expressed with ORF3-Myc in HEK293T cells. Transfected cell lysates were immunoprecipitated with anti-Myc antibody-conjugated beads and immunoblotted with anti-Myc and anti-Flag antibodies. We found that while the IKBKB kinase domain was dispensable for ORF3 association, ULD, SDD and NBD are required for IKBKB-ORF3 interaction (Figure 2). It is known that NEMO not only plays an essential role in IKBKB activation, but its binding to IKBKB is also required for efficient IKBKB activation and IkBα phosphorylation [3]. We thus examined whether ORF3 protein could interfere with NEMO-IKBKB interaction. As shown in Figure 3, ORF3 could not bind NEMO (Figure 3A) and, within the range of ORF3 concentrations (0.2, 0.4 and 0.8 mg/mL total proteins), it was not compete with NEMO to interact with IKBKB (Figure 3B). 

On the other hand, to identify the ORF3 region(s) responsible for IKBKB binding, three truncated ORF3 constructs were generated. These ORF3 variants include (1) ORF3 with deleted N terminus (lacking amino acids 1-73): (ΔN-ORF3), (2) ORF3 with deleted C terminus (lacking amino acids 174-227): (ΔC-ORF3) and (3) ORF3 with both N and C terminal regions deleted (ΔNC-ORF3). All three truncated ORF3 constructs were Myc-tagged at the C-terminus. Unfortunately, despite several attempts, we detected the minimal expression of the ΔC-ORF3-Myc and ΔNC-ORF3-Myc constructs (data not shown). The role of the C-terminus to viral protein degradation and its expression level have been reported elsewhere [14,15]. We speculated that amino acids residing in the C-terminus of ORF3 might be involved in protein translation/stabilization or protecting protein from degradation. We thus did not include them in further co-immunoprecipitation studies. The plasmid expressing ΔN-ORF3-Myc was co-transfected with a plasmid expressing IKBKB-Flag into HEK293T cells. The results show that ΔN-ORF3-Myc could interact with IKBKB-Flag, suggesting that the main interaction mediator resides in residues 74-224 of ORF3 were required for IKBKB binding (Figure 3C). 

### 2.3. PEDV ORF3 Protein Augments IKBKB—Mediated NF-ҡB Promoter Activation

**Figure 4:**
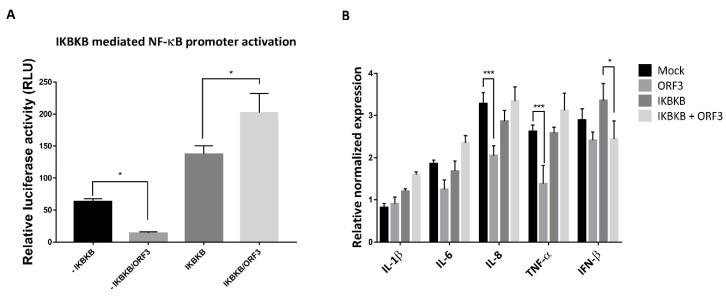
ORF3 increased the activation of IKBKB-meditated NF-κB promoter activity. (**A**) HEK293T cells were grown in 24-well plates and transfected with plasmids expressing ORF3, IKBKB, firefly luciferase driven NF-ҡB promoter and *Renilla* luciferase. At 24 hpt, cells were treated with 50 ng/mL TNF-α for 8 hrs. Cells were then lysed, and the firefly luciferase and *Renilla* luciferase activities were measured. Data (*n* = 3) were shown as the relative firefly luciferase activities normalized to the *Renilla* luciferase activities. *, *p* < 0.05. (**B**) LLC-PK1 cells were transfected with the plasmid expressing IKBKB or/and ORF3 for 24 h following with TNF-α treatment (50 ng/mL) for 8 hrs. Cells were lysed and subjected to total RNA extraction. The RT-qPCR was performed to detect the relative expression of cytokine mRNA. Relative mRNA expression (*n* = 3) of cytokine mRNA was normalized with the internal control β-actin. Error bars indicated mean± standard error of means of relative normalized mRNA expression from duplicate experiments. *, *p* < 0.05; ***, *p* < 0.001.

PEDV infection has been reported to be associated with NF-κB signaling pathways [16,17]. In particular, non-structural proteins and structural proteins including E and M, and ORF3 of PEDV were found to suppress PRDII promoter activation in an NF-κB-dependent manner [17]. This evidence has prompted us to hypothesize that PEDV ORF3 might also play a regulatory role in the NF-ҡB signaling pathway. To this end, we first investigated the effect of ORF3 overexpression on the IKBKB-mediated NF-ҡB promoter activity. As shown in Figure 4A, the activation of the NF-ҡB promoter was significantly upregulated in the presence of ORF3. Notably, upon TNF-α treatment, we found that the transcriptional levels of proinflammatory cytokines such as IL-8 and TNF-α were down-regulated in LLC-PK1 cells overexpressing ORF3 but not IKBKB compared to those of mock-transfected cells (Figure 4B). Although the NF-ҡB promoter activity was substantially elevated in the presence of both IKBKB and ORF3 (IKBKB/ORF3) (Figure 4A), we could not detect up-regulation of proinflammatory cytokine mRNA expression (Figure 4B). It is also worth noting that, in contrast to its effect on proinflammatory cytokine mRNA expression, ORF3 could significantly inhibit IKBKB-mediated IFN-β mRNA expression (Figure 4B). 

### 2.4. ORF3 Differently Regulated Type I IFN Production Mediated by IKBKB and RIG-I

**Figure 5:**
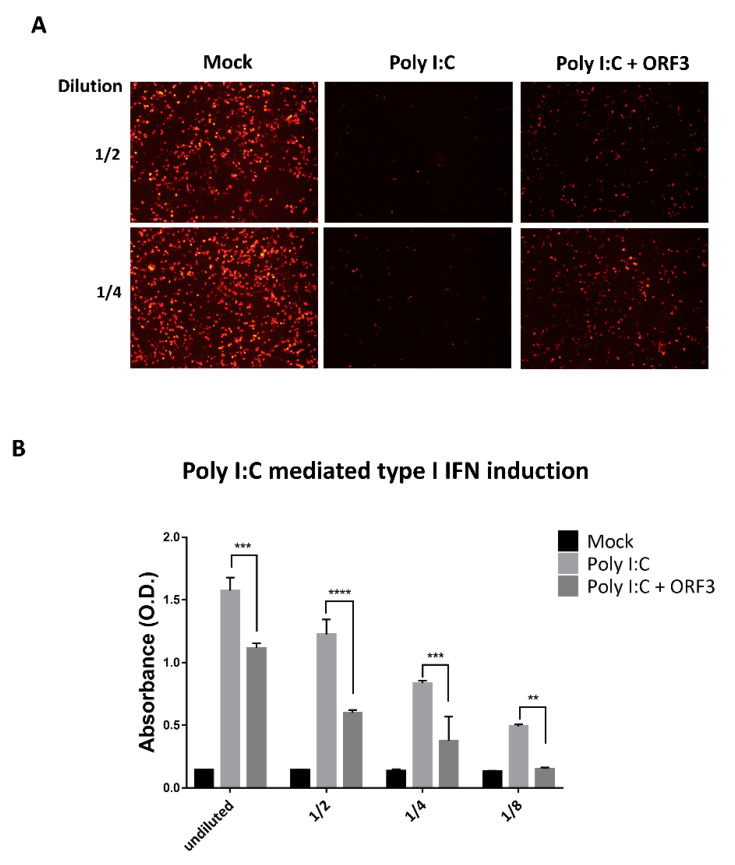
ORF3 inhibits Poly I:C mediated type I interferon production and induction. HEK293T cells were transfected with Poly I:C and ORF3. Cells transfected with empty plasmid were used as mock transfection. At 24 hpt, undiluted and serial 2-fold dilutions (1/2 and 1/4) of supernatants were collected and incubated in PK-15 cells for 24 hrs. After incubation, cells were infected with VSV-mCherry. Spreading of the virus in the cell monolayer was monitored by the detection of mCherry fluorescence with fluorescence microscopy (**A**). (**B**) Undiluted and serial 2-fold dilutions (1/2, 1/4 and 1/8) of supernatants (*n* = 3) were added into 96-well plate containing HEK-Blue^TM^ IFN-α/β cell suspension. After incubation for 24 hrs, induced HEK-Blue^TM^ IFN-α/β cell supernatants were used to measure type I IFN induction through JAK-STAT pathway activation by a secreted embryonic alkaline phosphatase (SEAP) detection following the manufacturer’s protocol. The SEAP levels were quantified using a spectrophotometer at 655 nm. Error bars indicated mean ± standard error of means of absorbance values. **, *p* < 0.01; ***, *p* < 0.001; ****, *p* < 0.0001.

The results showing that ORF3 could decrease IFN-β mRNA expression point to a possible role of ORF3 protein in type I IFN inhibition. We initially attempted to examine whether ORF3 suppressed Poly I:C induced type I IFN production. The culture supernatants harvested from Poly I:C or/and ORF3-transfected HEK293T cells were used to treat PK-15 cells, followed by infection with VSV–mCherry. While VSV–mCherry reporter virus could efficiently replicate in PK-15 cells pretreated with supernatants harvested from Poly I:C/ORF3-transfected cells, infected cells pre-treated with supernatants from Poly I:C transfected cells showed only marginal VSV–mCherry infection (Figure 5A). The same set of supernatants was then subjected to determine type I IFN activity on Human Embryonic Kidney (HEK)-Blue^TM^ IFN-α/β cells. These particular cells are specifically designed to monitor JAK-STAT pathway activation induced by the binding of type I IFN receptors to their ligands. This subsequent JAK-STAT pathway activation could be quantified by the detection of a reporter gene expressing a secreted embryonic alkaline phosphatase (SEAP) under the control of the ISG54 promoter. Supernatants from Poly I:C/ORF3-transfected cells had a low SEAP signal compared to that of Poly I:C-transfected cells (Figure 5B), suggesting that the ORF3 could inhibit Poly I:C-induced type I IFN production and induction. 

**Figure 6:**
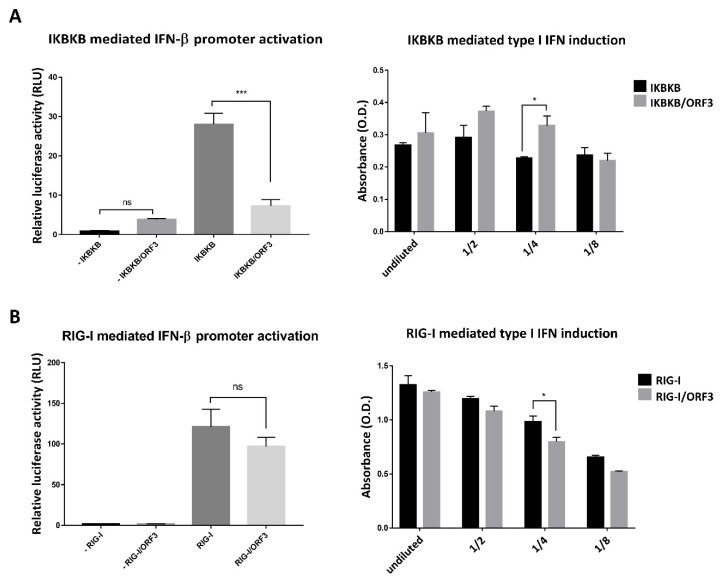
ORF3 regulates IKBKB or RIG-I mediated IFN-β promoter activation and type I IFN production via distinct pathways. (**A**) and (**B**) HEK293T cells were grown in 24-well plates and transfected with plasmids expressing ORF3, IKBKB or RIG-I, firefly luciferase driven IFN-β promoter and *Renilla* luciferase. At 24 hpt, undiluted and serial 2-fold dilutions (1/2, 1/4 and 1/8) of supernatants (n = 3) were collected and added into 96-well plate following with HEK-Blue^TM^ IFN-α/β cell suspension. After incubation for 24 hrs, induced HEK-Blue^TM^ IFN-α/β cell supernatant was used to measure type I IFN induction through JAK-STAT pathway activation by a SEAP detection following the manufacturer’s protocol. The SEAP levels were quantified using a spectrophotometer at 655 nm (A and B, right panels). Cells were lysed, and the firefly luciferase and *Renilla* luciferase activities were measured. Data were shown as the relative firefly luciferase activities normalized to the *Renilla* luciferase activities (A and B, left panels). *, *p* < 0.05; ***, *p* < 0.0001; ns, no statistical difference.

As the IKBKB/NF-ҡB signaling module has also been shown to induce type I interferon production [18], we next determined whether the ORF3 protein had any effects on IKBKB-mediated type I IFN production and induction. Plasmids expressing ORF3, IKBKB, firefly luciferase driven IFN-β promoter and Renilla luciferase were transfected into HEK293T cells. The luciferase signals were then measured by dual-luciferase assays to determine IFN-β promoter activity. The supernatants harvested from these transfected cells were also used to induce SEAP production from HEK-Blue^TM^ IFN-α/β cells as described above. To our surprise, while ORF3 was found to substantially inhibit IKBKB-mediated IFN-β promoter activation, it could trigger IKBKB-mediated type I IFNs induction (Figure 6A). We then asked whether ORF3 has a similar effect when one of the retinoic acid-inducible gene I (RIG-I) like receptor (RLRs) members, RIG-I was also co-expressed with ORF3 in cells monitoring IFN-β promoter activity. It is also noteworthy that while the ORF3 barely affected RIG-I mediated IFN-β promoter activity (Figure 6B, Left panel), supernatants harvested from RIG-I/ORF3-transfected cells could suppress RIG-I mediated type I IFN induction (Figure 6B, right panel). These results suggest that, in contrast to the activation via the IKBKB-mediated type I IFN pathway, ORF3 can down-regulate type I IFN induction through the RIG-I mediator.

## 3. Discussion

Innate immune response is typically activated soon after viral infection. However, many viruses have evolved mechanisms to antagonize host innate immune functions for the purpose of facilitating their efficient replication and infection. PEDV utilizes its many proteins to restrict a host antiviral state. For example, N protein was identified as an interferon (IFN) antagonist by interfering with interferon regulatory factor 3 (IRF3) and TANK-binding kinase 1 (TBK1) interaction and consequently inhibited IFN production [16]. Non-structural protein 5 (nsp5) or 3C-like protease disrupted RIG-I/melanoma differentiation-associated gene 5 (MDA5) signaling by cleaving NEMO, resulting in an inhibition of IFN induction [19]. PEDV nonstructural protein 1 (nsp1) suppressed IRF1-mediated IFN-λ response [20]. While very little is known about the role of PEDV sole accessory protein as a potential host immune response antagonist, ORF3 has been proposed as a virulent factor acting to subdue NF-κB and IRF1 promoter activation [17,20]. 

Our proteomics analysis of ORF3 interactome identified IKBKB and other cellular proteins involved in host immune responses [7]. As IKBKB acts as a central regulator in controlling key immune signaling pathways, it is of interest to initially examine whether ORF3 could interfere with the immune signaling pathway through IKBKB binding. IKBKB is a component of the IKK kinase complexes, which comprise two kinases (IKBKA and IKBKB) and a regulatory subunit, NEMO/IKKγ. Phosphorylation of IkBα by IKK complexes is required to break up the binding between IkBα and NF-κB, and subsequently to release NF-κB into the nucleus. Nuclear NF-κB binds to NF- κB promoter to regulate transcription of many genes participating in host immune and inflammatory responses [4,21]. In our study, we speculated that IKBKB could be one of the key host factors involved in restricting PEDV replication in VeroE6 cells. Although PEDV_AV12_-ORF3 replication was recovered in IKBKB-knock out cells, we cannot rule out the possibility that other PEDV proteins may also play a role in the reconstituted ability to replicate [16,19]. Vero cells are type I interferon-deficient [22,23], and thereby they are not responsive to IKBKB-induced type I IFN induction. However, in addition to regulation of type I IFN production, IKBKB as a master regulator of innate immunity takes part in various immune signaling pathways and other cellular mechanisms. Importantly, IKBKB has a direct role in the NF-кB signaling pathway which is essential for proinflammatory cytokine production and autophagy during virus infection [24]. For PEDV, the virus inhibited the induction of TNF-α in Vero and MARC-145 cells. There are several viral proteins identified to regulate NF-кB signaling pathway. For instance, nsp1 has been demonstrated to inhibit NF-κB-mediated proinflammatory cytokines, resulting in supporting virus propagation at an early time point [17]. We thus speculated that the overexpression of IKBKB mediates NF-кB activation and proinflammatory cytokine production to counteract with PEDV infection in Vero cells even in the absence of type I IFN production.

IKBKB contains an N-terminal kinase domain, a ubiquitin-like domain, an elongated a-helical scaffold/dimerization domain and a NEMO binding domain [13]. NEMO has also been known as an essential protein for IKBKB activation. NEMO binds to the NEMO binding domain of IKBKB for efficient phosphorylation of IκBα [3]. To examine the interacting domains of ORF3 and IKBKB, we performed co-immunoprecipitation assays and found that the IKBKB kinase domain alone was not sufficient to bind ORF3. It will be interesting to further investigate if IKBKB kinase activity is important for IKBKB and ORF3 association. In addition, as IKBKB and NEMO interaction could not be displaced by an increasing concentration of ORF3, we thus concluded that the observed interaction between ORF3 and NBD of IKBKB is inadequate to claim the ORF3’s role in competing with NEMO function. To this end, we speculated that the presence of ORF3 might interrupt or disturb IKBKB dimerization, resulting in the perturbation of downstream signaling cascade. 

Viral infection is one of the most potent stimuli, which trigger the NF-κB pathway through IKBKB activation. Viruses are capable of both inhibiting and activating NF-κB signal to support their productivity and pathogenesis. Accessory proteins from other coronaviruses, middle east respiratory syndrome coronavirus (MERS-CoV) 4a, 4b, and 8b have been revealed to inhibit MDA5- and TBK1-mediated NF-κB activation, respectively [25]. Also, severe acute respiratory syndrome coronavirus (SARS-CoV) 8a and 8ab bound IRF3 and accelerated IRF3 degradation through a ubiquitin-proteasome pathway, resulting in reduced IFN-β expression and facilitating virus replication [26]. Many viral proteins such as HBx protein of hepatitis B virus [27], nonstructural protein 3 (nsP3) of Venezuelan equine encephalitis virus (VEEV) [24] and vFLIP protein of Kaposi’s sarcoma-associated herpesvirus (KSHV) [28] have been reported to manipulate IKBKA or IKBKB and consequentially activate the NF-κB cascade. The aforementioned investigations on coronavirus accessory proteins and other viral proteins point to the potential roles of PEDV ORF3 protein in manipulating both NF-κB and type I IFN signaling. To investigate the effect of ORF3 overexpression on the IKBKB-mediated NF-ҡB promoter activity, cells were treated with TNF-α, a potent NF-ҡB stimulator. Optimized concentrations and durations of TNF-α treatment that triggered IkBα phosphorylation and provided distinguishable proinflammatory cytokine mRNA expression between experimental groups in HEK293T and LLC-PK1 cells were chosen for the study. Consistent with previous finding [17], our study showed that ORF3 inhibited NF-κB promoter activity [17] and down-regulated IL-8 and TNF-α mRNA expression. However, when IKBKB and ORF3 were co-expressed, NF-κB promoter activity was increased (Figure 4A, left panel), whilst IFN-β promoter activity was suppressed (Figure 6A, left panel). These observations prompt us to speculate that the IKBKB–ORF3 interaction might regulate NF-ҡB and type I IFN signaling pathways via distinct modes of action. 

In addition, we found that ORF3 was shown to elevate IKBKB mediated type I IFN induction (Figure 6A, right panel), but it had a robust negative effect against Poly I:C (Figure 5B) or RIG-I-mediated type I IFN induction ((Figure 6B, right panel). Despite the negative impacts ORF3 has on Poly I:C and RIG-I-mediated type I IFN induction, further experiments are still needed to pinpoint which type I IFN signaling intermediate(s) is targeted by ORF3. The discrepancy observed between NF-κB and type I IFN signaling pathways may reflect the importance of a fine balance in the relative amounts of signaling intermediates involved in signal transduction. The interplay between the key signaling proteins might be perturbed or disrupted by ORF3 in the context of PEDV infection. We also speculate that the differential regulation of PEDV ORF3 on both immune signaling pathways is a result of both direct and indirect interaction of ORF3 and IKBKB. Furthermore, it is likely that the ambiguous effects of ORF3 on both NF-κB and type I IFN inductions are due to the fact that IKBKB is able to interact with several other cellular signaling pathways [13]. Conversely, IKBKB could be one of the many other key transcription factors whose functions are also altered in the presence of ORF3. It is worth mentioning that although the HEK293T cells used in this study serve as a reliable transfection cell culture model, other porcine cell lines which are permissive and biologically relevant to PEDV infection such as IPEC-J2, a porcine jejunal cell line derived from a neonatal pig and its derivative (IPEC-DQ) [20], could be employed.

**Figure 7:**
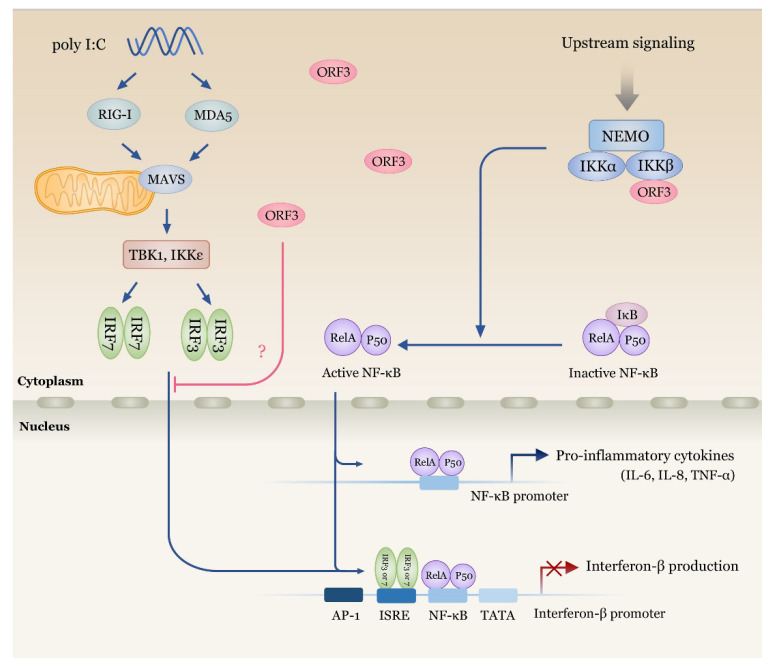
Schematic representation summarizing the impacts of PEDV ORF3 on NF-κB and IFN-β signaling cascades. ORF3 is capable of interacting with IKBKB (IKKβ) with no effect on IKBKB and NEMO binding. IKBKB-mediated NF-κB promoter activity is upregulated in the presence of ORF3. On the other hand, ORF3 significantly down-regulates IKBKB- and Poly I:C-mediated IFN-β promoter activity. Further experiments are still needed to verify which signaling intermediates of type I IFN induction pathway are the target of ORF3.

In conclusion, we identified an emerging role of PEDV ORF3 as a regulator of IKBKB mediated NF-κB and IFN-β promoter activation, proinflammatory cytokines expression and type I IFN signaling cascade. A schematic representation shown in Figure 7 summarizes the effects of ORF3 observed in our study on both innate immune signaling pathways. Our present study provides a possible molecular mechanism of ORF3 in manipulating and impeding host immune response and broadens our understanding of ORF3’s role in PEDV pathogenesis. While this study unveiled one of the functions of ORF3 in facilitating PEDV pathogenesis, further investigation is still needed to uncover an as yet unknown involvement of this accessory protein in other aspects of PEDV biology and cellular immune signaling pathways.

## 4. Materials and Methods 

### 4.1. Cell Lines and Viruses 

The African green monkey kidney cell line VeroE6 and human embryonic kidney 293T (HEK293T) cells were cultured in Opti-MEM^®^ (Gibco^®^, Invitrogen, Carlsbad, CA, USA) supplemented with 10% fetal bovine serum (FBS) at 37 °C in a humidified atmosphere of 5% CO_2_. Porcine cell line LLC-PK1 (ATCC^®^ CL-101™) was maintained in Medium 199 containing 1.5 g/L to 2.2 g/L sodium bicarbonate supplemented with 3% FBS at 37 °C in a humidified atmosphere of 5% CO_2_. PEDV_AV12__ORF3 [12] was propagated in VeroE6 cells in Opti-MEM^®^ containing recombinant trypsin (2 µg/mL) (Thermo Scientific, Waltham, MA, USA). The recombinant vesicular stomatitis virus (VSV) expressing the mCherry protein (VSV-mCherry) constructed in our laboratory was previously described [29]. 

### 4.2. Plasmid Construction

pCAGGS plasmids expressing NF-kappa-B essential modulator (NEMO) with hemagglutinin (HA)-tag at the N-terminus (HA-NEMO), an inhibitor of nuclear factor kappa-B kinase subunit beta with Flag-tag at the C-terminus (IKBKB-Flag) [7] were used as a template for making IKBKB domain deletion, as shown in Figure 2. All plasmids were subject to direct nucleotide sequencing (First Base, Selangor, Malaysia) and western blot analysis for protein expression. 

### 4.3. Dual-Luciferase Assay

HEK293T cells were plated in 24-well plates at a density of 3 × 10^5^ cells/well and co-transfected with the plasmids expressing firefly luciferase driven under NF-ҡB (pNF-Luc) or IFN-β promoter, IKBKB-Flag, ORF3-Myc and *Renilla* luciferase (as an internal control) and mock-transfected. At 24 h post transfection (hpt), cells were treated or untreated with TNF-α for 8 hrs, then lysed and subjected for dual-luciferase assay (Promega, Madison, WI, USA) following the manufacturer’s instructions. The data represented relative firefly luciferase activity normalized to *Renilla* luciferase activity. 

### 4.4. Real-Time PCR Amplification of Proinflammatory Cytokines

Total RNA was isolated from LLC-PK1 cells transfected with plasmids expressing ORF3-Myc or/and IKBKB-Flag by using RNeasy kit (Qiagen, Venlo, Netherlands) and then subjected to RT-qPCR using Luna^®^ Universal One-Step RT-qPCR Kit (NEB, Ipswich, MA, USA) according to the manufacturer’s protocol. RT-qPCR was performed using the CFX96 Touch™ Real-Time PCR Detection System (BioRad, Hercules, CA, USA). The housekeeping gene β-actin was used as an internal control. Normalized data from each sample relative to zero were compared by the threshold cycle (∆∆^CT^) method [30]. All primers targeting porcine proinflammatory cytokine mRNA included sTNF-α (F: 5′-AACCTCAGATAAGCCCGTCG-3′ and R: 5′-ACCACCAGCTGGTTGTCTTT-3′), sIL−6 (5′-CTGGCAGAAAACAACCTGAACC-3′ and R: 5′-TGATTCTCATCAAGCAGGTCTCC-3′), sIL−8 (F: 5’-CCGTGTCAACATGACTTCCAA–3’ and R: 5’-GCCTCACAGAGAGCTGCAGAA-3’), sIL−1β (F: 5’-ACCTGGACCTTGGTTCTCTG-3’ and R: 5’-CATCTGCCTGATGCTCTTGT-3’) and β-actin (F: 5’-ATCGTGCGTGACATTAAG-3’ and R: 5’-ATTGCCAATGGTGATGAC-3’.

### 4.5. Confocal Microscopy

The plasmids expressing IKBKB-Flag and ORF3-Myc were co-transfected into VeroE6 and LLC-PK1 cells using Fugene^®^ HD transfection reagent (Promega) following the manufacturer’s instructions. At 24 hpt, cells were fixed with 4% paraformaldehyde in PBS for 20 min at 4 °C. Subsequently, cells were washed three times with PBS and blocked with PBS containing 10% FBS, 1% BSA, and 0.2% TritonX-100 for 1 h. Cells were then incubated for 1 h with rabbit anti-Flag (Abcam, Cambridge, MA, USA) and mouse anti-Myc antibodies (Invitrogen, Carlsbad, CA, USA) diluted in 10% FBS at a dilution of 1:500 and 1:500, respectively. Goat anti-rabbit IgG Alexa Fluor 488 and anti-mouse IgG Alexa Fluor 647 antibodies (both from Abcam) in 10% FBS at a dilution of 1:1000 were used as secondary antibodies and further incubated for 1 h. The glass slips were mounted on slides with ProLong^TM^ Gold Antifade Mountant with DAPI (Invitrogen). The samples were analyzed by Fluoview^TM^ FV1000 confocal microscopy (Olympus, Tokyo, Japan).

### 4.6. Virus Infection

PEDV_AV12__ORF3 virus [12] was adsorbed onto VeroE6 cells grown in a 6-well plate. After incubation for 1 h, the cells were washed twice with PBS and maintained in Opti-MEM^®^ supplemented with recombinant trypsin (2 µg/mL) (Thermo Scientific). Cell supernatants were collected at indicated time points for virus titration (syncytium forming unit (SFU)/mL) as previously described [12]. Briefly, serial 10-fold dilutions of the virus were inoculated into VeroE6 cells grown in 6-well plates for 24 hpi. Cells were then fixed and subjected to alkaline phosphatase assay detecting system using mouse anti-PEDV N antibodies (Medgene, Brookings, SD, USA) and goat anti-mouse IgG alkaline phosphatase antibodies (Abcam). Numbers of syncytium were counted based on color formation after the addition of 1-Step™ NBT/BCIP Substrate Solution (Thermo Scientific).

### 4.7. Co-Immunoprecipitation

To confirm physical interaction between ORF3 and IKBKB, HEK293T cells were co-transfected with pCAGGS plasmids expressing ORF3-Myc and IKBKB-Flag or vice versa (ORF3-Flag and IKBKB-Myc for reverse co-immunoprecipitation). At 24 hpt, the cells were washed with cold phosphate buffer saline (PBS) and re-suspended in pre-cooled IP lysis buffer (Pierce^TM^, Thermo Scientific) supplemented with protease inhibitor cocktail (Thermo Scientific). Cell lysates were cleared by centrifugation at 14,000× g for 5 min at 4 °C. Cleared lysates were then incubated with anti-Myc agarose (Pierce^TM^, Thermo scientific) with gentle rocking overnight at 4 °C. Immunoprecipitates were washed with TBST buffer (25 mM Tris-HCl, 0.15 M NaCl, 0.05% tween 20, pH 7.2) and eluted in sodium dodecyl sulfate (SDS) sample loading buffer. The samples were subjected to SDS-PAGE and western blot.

For ORF3 and NEMO competition assay, each of plasmids expressing ORF3-Flag, IKBKB-Myc and HA-NEMO were transfected into HEK293T cells. At 24 hpt, cells were collected and lysed with pre-cooled IP lysis buffer (Pierce^TM^, Thermo Scientific). An equal amount of lysate containing IKBKB-Myc and HA-NEMO and varied amounts of lysate with ORF3-Flag was combined and subjected to immunoprecipitation using anti-Myc bead as described above. Immunoprecipitates were eluted in sample buffer followed by SDS-PAGE and western blot.

### 4.8. Western Blot Analysis

Cells were collected and re-suspended in the Pierce^TM^ mammalian cell lysis buffer (Pierce^TM^, Thermo Scientific). Protein samples were mixed with SDS sample loading buffer, loaded onto 10% or 12% polyacrylamide gel, and transferred to nitrocellulose membranes. Membranes were incubated with rabbit anti-Myc (Abcam) antibody, rabbit anti-Flag (Abcam) antibody and rabbit anti-HA antibody. Rabbit anti-IKBKB antibody (Abcam) was used to detect endogenous IKBKB. Mouse anti-β-actin (Cell signaling) was used to detect β-actin for internal controls. Goat anti-rabbit IgG-HRP (KPL, MA, USA) and anti-mouse IgG-HRP antibodies were used as secondary antibodies for chemiluminescence detection by ChemiDoc™ XRS+ imager (BioRad-).

### 4.9. IFN Bioassay

Levels of IFN secreted by transfected HEK293T cells were determined as previously described [31,32], with some modification. HEK293T cells seeded in 6-well-plates one day before transfection to reach 80%–90% confluence. These cells were either mock-treated or transfected with ORF3, IKBKB, a combination of ORF3 and IKBKB expressing plasmids, and poly I:C by Fugene^®^ Transfection Reagent (Promega). Following transfection, cells were incubated in Opti-MEM^®^ containing 10% FBS, and supernatants were harvested 24 h post infection (hpi). PK-15 cells were seeded in 96-well plates one day before supernatant harvest. These PK-15 cells were then incubated with the harvested supernatants for 24 hours. The preincubated PK-15 cells were then infected with VSV-mCherry (MOI = 1). Cells expressing mCherry were visualized by fluorescence microscopy at 16 hpi. The presence of IFN in the supernatants was estimated based on their abilities to induce an antiviral state in PK-15 cells and inhibit VSV–mCherry replication, compared with known amounts of recombinant porcine IFN. 

### 4.10. ISRE/SEAP Activity Quantification

Type I IFNs in supernatants harvested from transfected HEK cells were quantified by HEK-Blue^TM^ IFN-α/β cells (Invivogen, San Diego, CA, USA) containing interferon-sensitive response element (ISRE) promoter. The presence of type I IFNs in supernatant induces ISRE promoter activation, which could be evaluated by using the substrate Qanti-blue (Invivogen) according to the manufacturer’s recommendations to measure secreted embryonic alkaline phosphatase (SEAP) activity. As a positive control, HEK-Blue^TM^ IFN-α/β cells were treated for 24 hours with supernatant of Poly I:C transfected HEK cells. 

### 4.11. Statistical Analysis

All data were expressed as means ± standard error of means (SEM). The differences in mean values of between groups were analyzed by two-way ANOVA. *p* values of <0.05 were considered statistically significant. GraphPad Prism 5.0 (GraphPad Software Inc., La Jolla, CA, USA) was used for statistical analyses.
